# The relevance of task-irrelevant sounds: hemispheric lateralization and interactions with task-relevant streams

**DOI:** 10.3389/fnins.2013.00264

**Published:** 2013-12-27

**Authors:** Ana A. Amaral, Dave R. M. Langers

**Affiliations:** ^1^International Neuroscience Doctoral Programme, Champalimaud Neuroscience Programme, Champalimaud Centre for the UnknownLisbon, Portugal; ^2^Department of Otorhinolaryngology/Head and Neck Surgery, University Medical Center Groningen, University of GroningenGroningen, Netherlands; ^3^National Institute for Health Research, Nottingham Hearing Biomedical Research Unit, School of Medicine, University of NottinghamNottingham, UK

**Keywords:** auditory cortex, dichotic listening, lateralization, cross-modal inhibition, Humans, fMRI

## Abstract

The effect of unattended task-irrelevant auditory stimuli in the context of an auditory task is not well understood. Using human functional magnetic resonance imaging (fMRI) we compared blood oxygenation level dependent (BOLD) signal changes resulting from monotic task-irrelevant stimulation, monotic task-relevant stimulation and dichotic stimulation with an attended task-relevant stream to one ear and an unattended task-irrelevant stream to the other ear simultaneously. We found strong bilateral BOLD signal changes in the auditory cortex (AC) resulting from monotic stimulation in a passive listening condition. Consistent with previous work, these responses were largest on the side contralateral to stimulation. AC responses to the unattended (task-irrelevant) sounds were preferentially contralateral and strongest for the most difficult condition. Stronger bilateral AC responses occurred during monotic passive-listening than to an unattended stream presented in a dichotic condition, with attention focused on one ear. Additionally, the visual cortex showed negative responses compared to the baseline in all stimulus conditions including passive listening. Our results suggest that during dichotic listening, with attention focused on one ear, (1) the contralateral and the ipsilateral auditory pathways are suppressively interacting; and (2) cross-modal inhibition occurs during purely acoustic stimulation. These findings support the existence of response suppressions within and between modalities in the presence of competing interfering stimuli.

## Introduction

The auditory system relies on various clues to segregate concurrent sound streams. These among others include clues related to sound source location, derived from head-related transfer functions, binaural interaural time differences, and interaural level differences, for instance (Ehret and Romand, 1997; Moore et al., 2010). The relationship between the lateralization of sound that is detected by the two ears and the lateralization of sound-evoked brain responses in the two hemispheres has been well studied. Both ears are known to project to both auditory cortices through contralateral and ipsilateral auditory pathways. Contralateral connections are more numerous than the ipsilateral ones (Rosenzweig, 1951; Hall and Goldstein, 1968; Reite et al., 1981). Brain responses resulting from monotic stimulation are bilateral and stronger in the hemisphere contralateral to stimulus presentation (Rosenzweig, 1951; Reite et al., 1981; Pantev et al., 1986; Scheffler et al., 1998; Alho et al., 1999; Woldorff et al., 1999; Langers et al., 2005; Della Penna et al., 2007). Furthermore, it has been shown using functional magnetic resonance imaging (fMRI) (Scheffler et al., 1998; Jäncke et al., 2002; Krumbholz et al., 2005) and magnetoencephalography (MEG) (Pantev et al., 1986; Fujiki et al., 2002; Kaneko et al., 2003) that the responses are sub-additive, that is, the sum of brain responses to left and right monotic stimulation exceeds the response to dichotic stimulation, a phenomenon known as 'binaural interaction'. To explain this, it has been suggested that a competition arises between the two pathways causing the stronger contralateral pathway to suppress the ipsilateral one, decreasing the overall brain responses (Fujiki et al., 2002; Kaneko et al., 2003; Brancucci et al., 2004; Della Penna et al., 2007).

Competition between inputs from the ipsi- and contralateral ear has been observed in the context of dichotic listening tasks, where participants are requested to attend to both ears receiving task-relevant streams and report the stimulus that was best heard. However, because dichotic stimulation typically involves multiple stimulus streams, attention forms an important confounding factor. Attention is known to influence auditory information processing (Jäncke et al., 1999; Petkov et al., 2004; Fritz et al., 2005; Rinne et al., 2005, 2009; Polley et al., 2006), and it can modulate neural responses in a “top-down” fashion (Kastner et al., 1999; Kastner, 2000; Fu et al., 2001). A common characteristic among some dichotic listening experiments is that subjects distribute their attention across the two presented streams (*divided attention*) and, when required, they are free to report from any ear (*non-forced attention*). Giving subjects the freedom to choose where to attend adds variability to these experiments: attentional shifts between ears are likely to occur and may interfere with lateralization effects related to “bottom up” acoustic clues. In electroencephalography (EEG) research the amplitude of the N1 component of the auditory event-related potential (ERP) that is evoked by the auditory stimuli is larger when the stimuli are attended than when the stimuli are unattended (Picton et al., 1971; Hillyard et al., 1973; Woldorff et al., 1993), but only if subjects are able to sustain their attention to the relevant stimuli (Donald and Young, 1982). In summary, it is unclear if the competition arising between the contralateral and ipsilateral pathways results from a bottom-up acoustic process, a top-down cognitive and attentional mechanism, or both.

In order to elucidate this, a change to the dichotic listening task can be introduced (Bryden et al., 1983) by forcing subjects to focus on the information presented to one of the two ears (*focused attention*) according to a provided instruction (*forced attention*). Thus, direction of attention becomes a controlled parameter in the experiment. Knowing a priori which task needs to be performed enables a person to focus on particular modalities, stimuli or stimulus features. Focusing on a task introduces a bias toward the stimuli or modalities that are relevant for task performance. However, the presence of task-irrelevant distracting stimuli can cause interference, which can result in unintended shifts of attention and consequently decreased performance or increased reactions times (Berti and Schröger, 2001). Recent EEG studies showed that the N1 amplitude is reduced in the presence of competing task-irrelevant auditory distractions presented to an unattended ear, when attention is directed to a task-relevant stream simultaneously presented to the other ear (Ahveninen et al., 2011; Ponjavic-Conte et al., 2012). This suggests that the presence of distractions interferes with top-down attentional enhancement of task-relevant stimuli.

A number of studies investigating the neural processing of task-irrelevant unattended stimuli showed that it may involve early sensory or later cognitive stages (Berti and Schröger, 2001; Sætrevik and Hugdahl, 2007; Sætrevik and Specht, 2009; Sabri et al., 2013). A recent study introduced a modified version of the dichotic listening paradigm with attentional instruction in which the relative intensity of the presented stimuli in both ears was varied (Westerhausen et al., 2009, 2010). This allowed not only the manipulation of a top-down cognitive cue (the instruction which ear to attend) but also a bottom-up acoustic cue (the interaural level difference). This study found that bottom-up and top-down mechanisms do not act independently. The authors identified two networks responsible for the interaction of the two different processes—a medial-lateral frontal cognitive control and a fronto-parietal attention control network. Moreover, in agreement with other studies (Barch et al., 1997; Duncan and Owen, 2000), they showed increases in the activations in frontal and parietal areas known to be involved in control of attention, indicating that degradation of the sensory input increases task difficulty that can be compensated with increased attention. However, interestingly, the study by Westerhausen et al. (2010) did not reveal any changes in activation in the auditory cortex (AC) nor a significant effect of stimulus manipulation.

The nature and mechanisms underlying the interactions between contralateral and ipsilateral auditory pathways remain an open question. In particular it is not known how these interactions change in the presence of differently attended or unattended stimulus streams. Research focusing on the role of task-irrelevant stimuli in auditory processing can be particularly relevant to increase our understanding of attentional disorders such as ADHD (e.g., Cherkasova and Hechtman, 2009), and conditions like tinnitus, also known as 'ringing in the ears', where subjects perceive sounds unrelated to their acoustical environment (e.g., Roberts et al., 2013).

In the present study, we used a forced attention dichotic listening task and varied the instruction and the task-irrelevant unattended stimulus identity, while maintaining an identical attended stimulus stream. This enabled us to modulate the top-down attentional processing and the bottom-up acoustic responses in relation to the processing of unattended stimuli. We used fMRI to test the hypothesis that unattended stimuli are essentially processed in bottom-up fashion, without top-down enhancement.

## Methods

### Subjects

Twenty-one healthy subjects (11 female, 2 left handed: one female and one male) aged between 20 and 61 (mean 40.4 ± 11.1 *SD*) years were recruited through advertising. All subjects reported normal hearing, which was verified through standard pure tone audiometry. Averaged over both ears, mean thresholds across octave frequencies from 0.25 to 2 kHz equaled 6.3 ± 7.0dB HL. All subjects had normal, or corrected-to-normal, vision. Each subject gave written informed consent in approved accordance with the guidelines of the Medical Ethical Committee of the University Medical Center Groningen in The Netherlands. This work is part of a bigger study in which subjects participated on two separate days. The present report concerns one of the two 1-h neuroimaging sessions that was preceded by an approximately half and hour instruction and practice session.

### Task and stimuli

The stimuli that were used in the neuroimaging session were letters (8 consonants: “L,” “T,” “R,” “C,” “H,” “K,” “S,” “Q”) spoken by a Dutch speaker as consonant-vowel or vowel-consonant utterances (/εl/,/te:/,/εr/,/se:/,/ha:/,/ka:/,/εs/, and/ky/, respectively). These were presented at a fixed rate of 1 Hz through MR-compatible headphones (MR Confon GmbH, Magdeburg, Germany; Baumgart et al., 1998).

**Figure 1:**
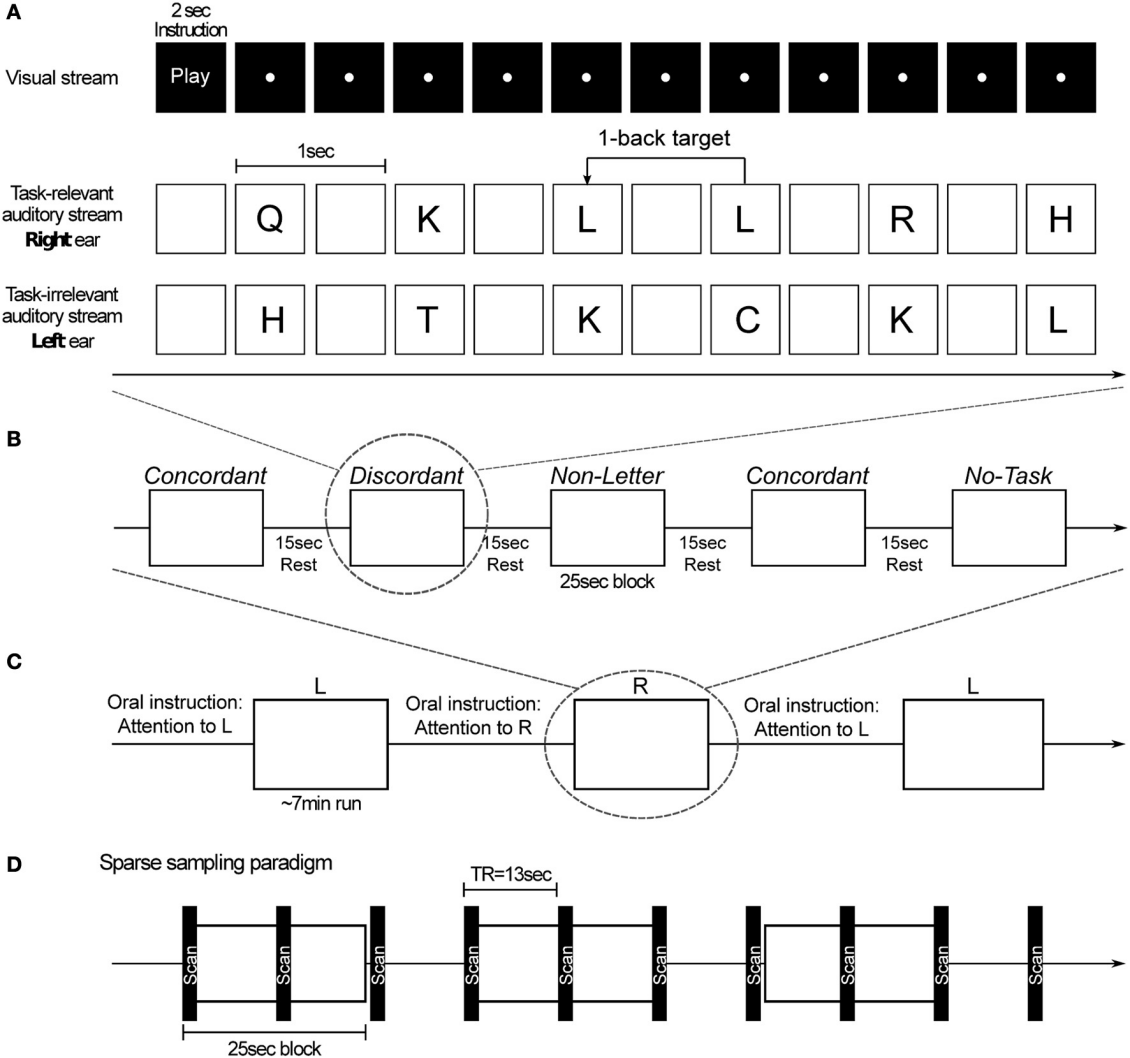
**(A)** Example of the 1-back task for a discordant block. For this condition, a task-relevant stream and a task-irrelevant stream were presented. In the beginning of each block subjects are visually instructed to perform the task or to listen passively. For each presentation of the task-relevant stimulus a button must be pressed: one button if the stimulus is different from the previous one and a different button if it is the same (target). **(B)** Illustration of a run's block-sequence with its experimental parameters. **(C)** Illustration of a sequence of runs. Before each run subjects are orally instructed to attend to one of the ears and perform the task (in all task blocks except *No-Task*) with the stimuli presented in that ear. **(D)** Functional MRI sparse sampling paradigm employed in the experimental procedure.

Subjects performed an auditory one-back task in which a task-relevant stream was presented in either the left or the right ear and, at the same time, a task-irrelevant stream was presented in the other ear. Both streams were spoken by different talkers, a female voice for the task-relevant stream and a male voice for the task-irrelevant stream. Subjects were required to attend to the task-relevant stream, compare consecutive stimuli, and press, at every stimulus presentation, one button if the stimuli were the same (i.e., a target), and a different button if the stimuli were different. Target stimuli were present at 30% probability. Subjects were instructed to answer as quickly and accurately as possible. All subjects' button presses were recorded. (Figure 1).

The stimuli that were presented in the task-irrelevant stream could either consist of the same letter as that in the target stream, a different letter, or it could consist of something different from a letter; in the latter case the competing stimuli comprised bird song syllables (Joly et al., 2012). The length of each of the auditory stimuli (letter and non-letter) ranged from 350 to 450 ms. During the dichotic conditions the left and right ear stimulus onset was synchronous. In addition, two monotic control conditions were included in which only one stimulus stream was presented. In one, the subjects performed the one-back task in the absence of a task-irrelevant stream; the other was a passive listening condition. This resulted in a total of five different conditions: *Concordant, Discordant, Non-Letter, No-Distractor*, and *No-Task*.

The neuroimaging session comprised six runs of approximately 7 min each. Before each run subjects were orally instructed to pay attention to the target stream in either the left (L) or the right (R) ear. Stimuli were presented in a block design. Each run comprised 10 blocks, two of each condition. Each block started with a 2-s visual instruction informing the subjects whether they had to perform the one-back task (for *Concordant, Discordant, Non-Letter* and *No-Distractor*) or not (for *No-Task*), followed by 23 stimulus presentations. Consecutive blocks were separated by 15 s during which no stimuli were presented. The order of the runs, conditions and stimuli was randomized. Subjects were instructed not to close their eyes (except for blinking) and fixate on a white dot on a screen during all runs (Figure 1).

The percentage of correct responses to stimulus presentations (number of correct “same” or “different” responses divided by the number of trials) was determined as a measure of subjects' performance. In order to avoid any masking effects of scanner noise we discarded all trials that coincided with acquisitions. Subjects' responses were considered to belong to a stimulus if a button was pressed between 100 and 1100 ms after its onset of presentation.

Performance was analyzed by means of a Two-Way repeated measures analysis of variance (ANOVA) with factors for attended ear (2 levels: L, R) and condition (4 levels: *Concordant, Discordant, Non-Letter, No-Distractor*; since *No-Task* did not produce behavioral results).

### Functional MRI

Neuroimaging was performed using a Philips Intera 3-Tesla MR system, equipped with an 8-channel phased-array (SENSE) head coil, at the Neuroimaging Center (NiC) in Groningen. An anatomical T1-weighted image was acquired for each subject before the functional imaging acquisition. Blood oxygenation level dependent (BOLD) images were acquired using a silent sparse sampling paradigm (TR = 13 s, TA = 2.0 s, which resulted in a silent interval of 11 s) to avoid interference from acoustic scanner noise (Hall et al., 1999). For each of the six runs, 32 dynamic T2^*^-sensitive echo planar imaging volume acquisitions were collected (TE = 22 ms, FOV = 192 × 192 × 144mm^3^, 64 × 62 × 48 matrix). All subjects wore earplugs to attenuate MRI-related gradient-induced noise.

Data were preprocessed with the Statistical Parametric Mapping software package (SPM8, FIL Welcome Trust Centre for Neuroimaging, London, UK) running in MATLAB® (Natick, Massachusetts: The MathWorks Inc.). The first dynamical scan in each run was used to trigger sound presentation but was excluded from the analyses due to lack of magnetization equilibrium. Each subject's data were realigned, the anatomical images were coregistered to the functional images, all images were normalized into Montreal Neurological Institute (MNI) stereotaxic space and smoothed using an isotropic 5-mm full-width at half-maximum Gaussian kernel. A logarithmic transformation was applied to express all voxels' signals in units of percentage signal change relative to the mean. A general linear model (GLM) was constructed for each subject that included 10 regressors modeling all experimental conditions (2 ears × 5 tasks), six regressors containing the estimated motion parameters modeling residual motion effects, and four regressors for each run describing a 0th to 3rd order Legendre polynomial modeling baseline and scanner drift effects. All statistical parametric maps resulting from constructed contrasts were thresholded at *p* < 0.05, corrected for family-wise errors (FWE), unless stated otherwise.

Anatomically defined regions of interest (ROI) were obtained from the WFU_PickAtlas software toolbox (Lancaster et al., 1997, 2000; Maldjian et al., 2003). All the areas from the Brodmann area (BA) atlas based on the Talairach Daemon database were used as ROIs. In addition, the following sensory ROIs were defined and separated into subvolumes in the left and right hemisphere for further analysis: primary auditory cortex (PAC: L-BA41+42 and R-BA41+42), secondary auditory cortex (SAC: L-BA22 and R-BA22), primary visual cortex (PVC: L-BA17 and R-BA17) and secondary visual cortex (SVC: L-BA19 and R-BA19).

For the aforementioned ROIs, the estimated regression coefficients were averaged across all voxels. Effects of interest were assessed by means of a Two-Way repeated-measures ANOVA comprising a 2-level factor ear (L or R) and a 5-level factor condition (*Concordant, Discordant, Non-Letter, No-Distractor* or *No-Task*). In the cases where the assumption of sphericity was violated the degrees of freedom were adjusted using Greenhouse-Geisser correction. *Post-hoc* analysis was performed using pairwise comparisons between conditions. A correction for multiple comparisons was performed using False Discovery Rate (FDR) criteria, controlled at 0.05 level. The following families of null-hypotheses were assessed by means of Student *t*-tests: stimulus effect (*No-Task* vs. baseline); task effect (*No-Distractor* vs. *No-Task*); distractor effect (*Concordant* vs. *No-Distractor*; *Discordant* vs. *No-Distractor*; *Non-Letter* vs. *No-Distractor*); distractor comparison (*Concordant* vs. *Discordant*; *Concordant* vs. *Non-Letter*; *Discordant* vs. *Non-Letter*); instruction effect (L-*No-Task* vs. R-*No-Task*; L-*No-Distractor* vs. R-*No-Distractor*; L-*Concordant* vs. R-*Concordant*; L-*Discordant* vs. R-*Discordant*; L-*Non-Letter* vs. R-*Non-Letter*).

## Results

### Behavior

**Figure 2:**
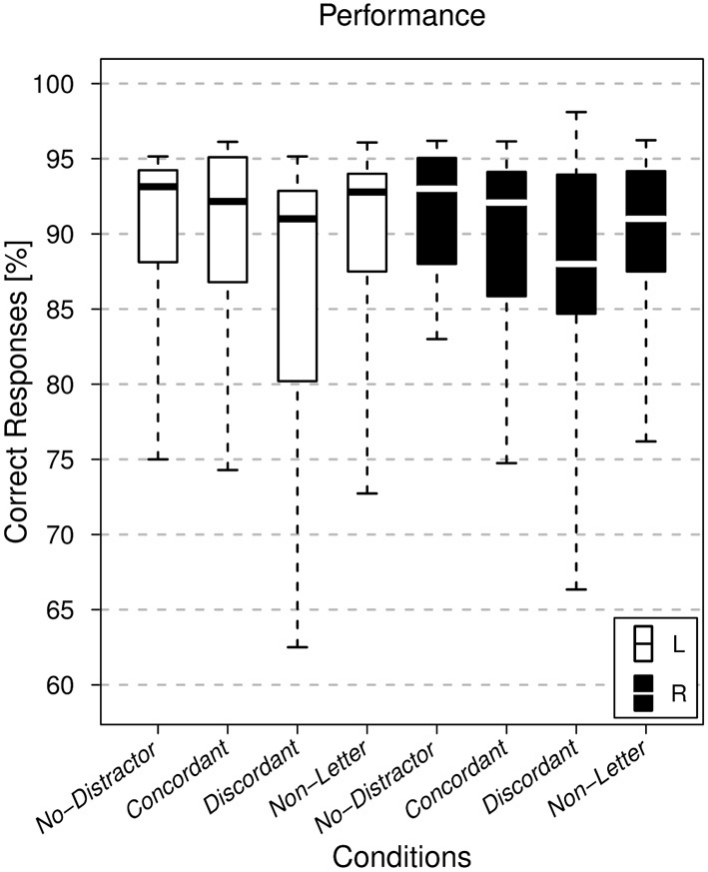
**Performance.** Boxplot showing the distribution of the individual subjects' performance, comparing the various task conditions. Conditions corresponding to the attended left ear (L, white) and attended right ear (R, black) runs are shown. The horizontal line within each box represents the median.

As shown in Figure 2, task performance was high for all subjects. The mean percentage of correct responses over all subjects was above 85% for each combination of ear and condition. The ANOVA did not reveal any significant dependence upon the factor ear (*p* = 0.949). However, for the factor condition there was a significant dependence (*p* = 5 × 10^−6^). Correct responses to the discordant letter condition were lowest, followed by the non-letter and concordant conditions. Since the interaction between both factors was not significant (*p* = 0.93), results were averaged over the non-significant factor ear and a paired Wilcoxon signed-rank test was calculated for all pairs of conditions, and FDR corrected for multiple comparisons (controlled at 0.05 level). Significant differences were found for all three pairs involving the discordant letter condition: *No-Distractor* vs. *Discordant* (*p* = 2.2 × 10^−3^), *Concordant* vs. *Discordant* (*p* = 4.0 × 10^−5^), *Non-Letter* vs. *Discordant* (*p* = 3.5 × 10^−3^). The *Concordant* vs. *Non-Letter, No-Distractor* vs. *Non-Letter* and *No-Distractor* vs. *Concordant* comparisons did not reach statistical significance (*p* > 0.1).

### fMRI contrasts

**Figure 3:**
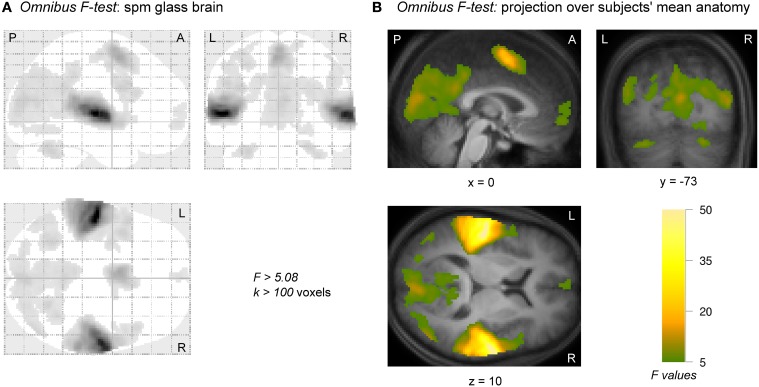
**Activation and deactivation to all conditions relative to baseline according to an omnibus *F*-test.** Activation clusters were observed in right and left auditory cortices. Visual and motor-related areas were also active as was a small region in frontal cortex. **(A)** A “glass brain” image, showing the thresholded activation across the entire brain. **(B)** Activations of nine adjacent slices were overlaid over the subjects' mean anatomical image. Images were thresholded at a confidence level *p* < 0.05 (FWE-corrected) and cluster size *k* > 100 voxels. A, anterior; L, left; P, posterior; R, right.

The significance of the (de)activation to all 10 conditions relative to baseline according to an omnibus *F*-test is shown in Figure 3. Activation was found in widespread areas of the brain: sensory auditory and visual cortices in the temporal and occipital lobes, motor and pre-motor areas, as well as regions in the frontal lobe.

**Figure 4:**
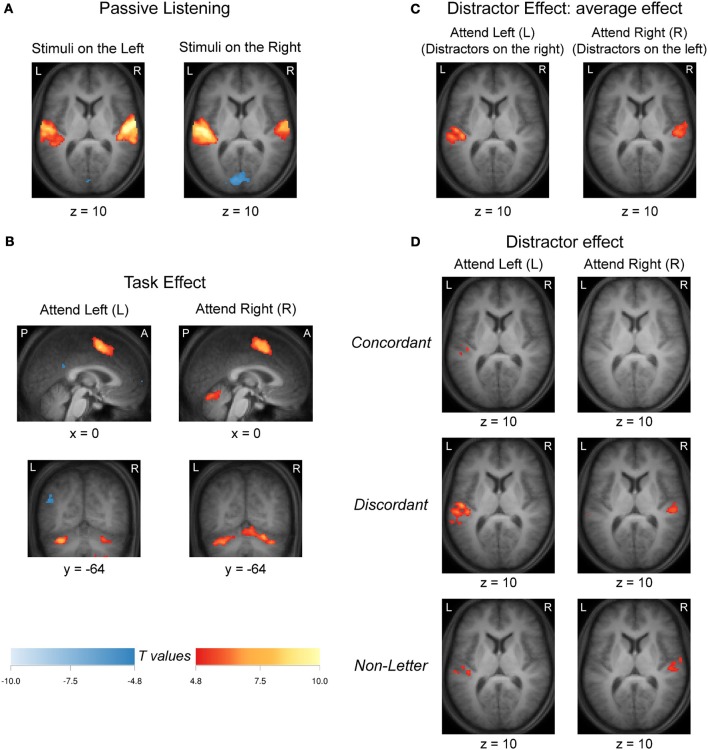
**(A)** Activation (deactivation) for the passive listening condition (*No-Task*) relative to the silent baseline for left (L) and right (R) ear stimulus presentations. **(B)** Task related activation (deactivation) derived from the contrast *No-Distractor* vs. *No-Task*. **(C)** Activation (deactivation) for the average distractor effect relative to task condition [(*Concordant* + *Discordant* + *Non-Letter*)/3 vs. *No-Distractor*] when attending the left (L) and right (R) ear. **(D)** Activation (deactivation) for each distractor effect relative to task condition (*Concordant* vs. *No-Distractor*; *Discordant* vs. *No-Distractor*; *Non-Letter* vs. *No-Distractor*) when attending the left ear (L) and right ear (R). All images were thresholded at a confidence level *p* < 0.05 (FWE-corrected). Activations (deactivations) of nine adjacent slices were overlaid over the mean anatomical image. Hot (cold) colors refer to increased (decreased) signals. A, anterior; L, left; P, posterior; R, right.

Group-level activation to passive listening (*No-Task*), contrasted against baseline (silence), for the L and R ear presentations is shown in Figure 4A. For each contrast there was bilateral activation in the auditory cortex that was stronger in the hemisphere contralateral to the stimulated ear. In addition, bilateral decreased signals relative to the baseline of the medial visual cortex in the calcarine sulcus was observed.

The effect of task performance, addressed through a *No-Distractor* vs. *No-Task* contrast, is shown in Figure 4B. Both when instructed to attend the left (L) or right (R) ear, activation was found in the supplementary motor area and cerebellum.

To study the effect of the task-irrelevant stimuli, contrasts were computed for the three respective conditions (*Concordant, Discordant*, and *Non-Letter*) against the condition without any task-irrelevant stream (*No-Distractor*), for the left and right ear presentations separately. Figure 4C shows the mean effect of all three conditions when presenting task-irrelevant information to the right ear when attending the left ear, or to the left ear when attending the right ear. Unilateral activation was observed in the auditory cortex contralateral to the irrelevant stimuli (that is, ipsilateral to the attended ear). Figure 4D further shows the contrasts involving each of these three conditions separately. As before, the effect was present on the side contralateral to the presentation of the task-irrelevant stimuli. The most extensive activation was observed in the contrast involving the discordant letters (i.e., *Discordant* vs. *No-Distractor*). The non-letter stimuli resulted in more confined activation (i.e., *Non-Letter* vs. *No-Distractor*), although activation still peaked in similar locations. Finally, the concordant letters (i.e., *Concordant* vs. *No-Distractor*) evoked the least extensive activation (or no significant effect at all when attending the right ear).

**Figure 5:**
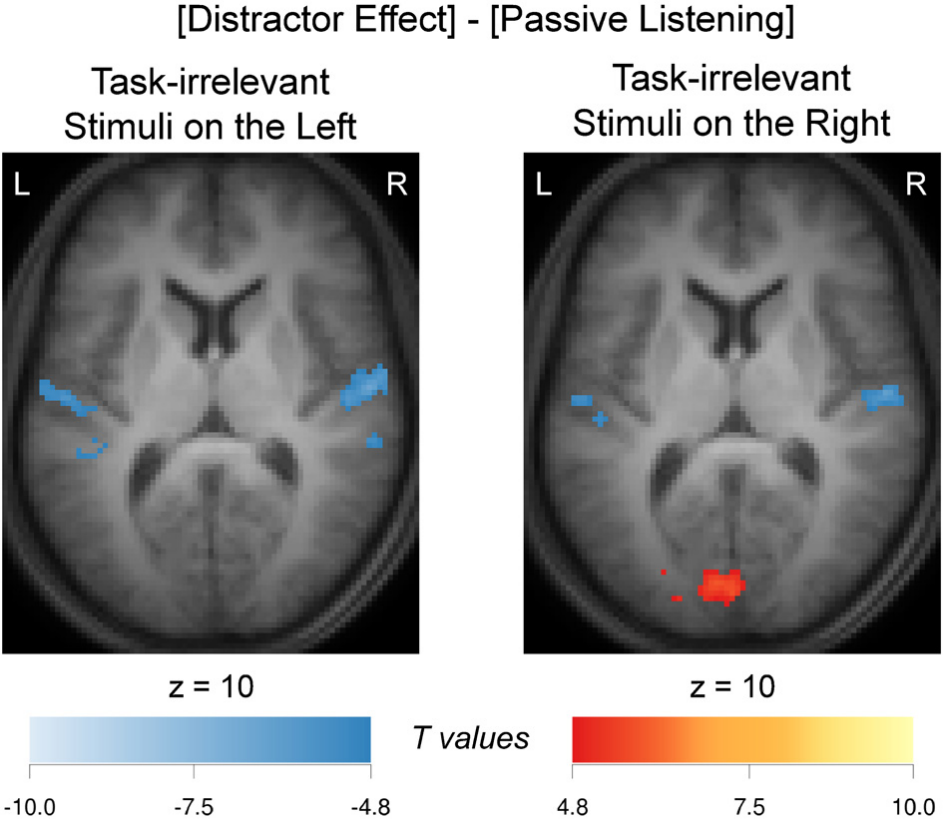
**Distractor effect and passive-listening comparison.** Supra-additivity (sub-additivity) resulting from comparison of averaged distractor activation maps (Figure 4C) and passive listening (Figure 4A), that is ([(*Concordant* + *Discordant* + *Non-Letter*)/3—*No-Distractor*] vs. *No-Task*), when these task-irrelevant stimuli were presented on the left ear and on the right ear. Images were thresholded at a confidence level *p* < 0.05 (FWE-corrected). Supra-additivity (sub-additivity) of nine adjacent slices were overlaid over the mean anatomical image over all subjects. Hot (cold) colors refer to increased (decreased) signals. L, left; R, right.

In order to assess whether the apparent differences in activation patterns in Figure 4C and Figure 4A were significant, a comparison between these two contrasts was made. The resulting contrast [(*Concordant* + *Discordant* + *Non-Letter*)/3 − *No-Distractor*] vs. *No-Task* is shown in Figure 5. When comparing the activation evoked by a task-irrelevant stream in the left ear (when attending to the right ear in a task condition *with* distractors) to the activation evoked by a single task-irrelevant stream in the same left ear (during passive listening *without* distractors), bilateral decreased responses in the auditory cortex were observed. A similarly bilateral but less extensive decreased response pattern was observed when comparing the activation evoked by a task-irrelevant stream in the right ear (when attending to the left ear) to the activation evoked by a single task-irrelevant stream in the same right ear (during passive listening). That is, bilateral auditory cortex responded significantly less strongly to a task-irrelevant stream that was presented to one ear in the presence of a task-relevant stream in the other ear than to a task-irrelevant stream that was presented alone in one ear. In other words, the activations evoked by the task-relevant and task-irrelevant streams combine sub-additively in both hemispheres. Figure 5 further shows activation of the primary visual cortex in the calcarine sulcus, which was significant only for the comparison concerning the activation evoked by task-irrelevant streams presented to the right ear.

### ROIs

All Brodmann areas that showed significance for any factor in the ANOVA are presented in Table 1. The pattern of brain areas that were responsive to the stimuli and task well agreed with those according to the voxel-wise omnibus test in Figure 3. Interactions between ear and condition were never significant.

**Table 1:** **Significance of ROI activation for one or both of the factors defined in the ANOVA statistics, ear and condition**.

**Brodmann area**	**Ear**	**Condition**
1 (Intermediate postcentral area)	n.s.	^*^
2 (Caudal postcentral area)	n.s.	^*^
5 (Preparietal area)	n.s.	^*^
6 (Agranular frontal area)	n.s.	^***^
19 (Preoccipital area)	n.s.	^***^
22 (Superior temporal area)	n.s.	^***^
29 (Granular retrolimbic area)	n.s.	^***^
30 (Agranular retrolimbic area)	n.s.	^**^
31 (Dorsal posterior cingulate area)	n.s.	^***^
36 (Ectorhinal area)	n.s.	^*^
39 (Angular area)	^*^	^***^
40 (Supramarginal area)	n.s.	^**^
41 (Anterior transverse temporal area)	n.s.	^***^
42 (Posterior transverse temporal area)	n.s.	^***^
43 (Subcentral area)	n.s.	^***^
44 (Opercular area)	n.s.	^***^
45 (Triangular area)	n.s.	^***^
**Sensory ROI^a^**	**Ear**	**Condition**
L-PAC (Left primary auditory cortex)	n.s.	^***^
R-PAC (Right primary auditory cortex)	^**^	^***^
L-SAC (Left secondary auditory cortex)	n.s.	^***^
R-SAC (Right secondary auditory cortex)	n.s.	^***^
L-PVC (Left primary visual cortex)	n.s.	n.s.
R-PVC (Right primary visual cortex)	n.s.	n.s.
L-SVC (Left secondary visual cortex)	n.s.	^**^
R-SVC (Right secondary visual cortex)	n.s.	^***^

All Brodmann areas (BA) were tested; areas that are not listed showed no significant effect for either factor. The sensory ROIs that were defined for further analysis are all included as well.

* p < 0.05;

** p < 0.01;

*** p < 0.001; n.s., non-significant. BA labels according to Strotzer (2009).

a Greenhouse-Geisser corrected significance of the two factors is reported for the sensory ROIs.

**Figure 6:**
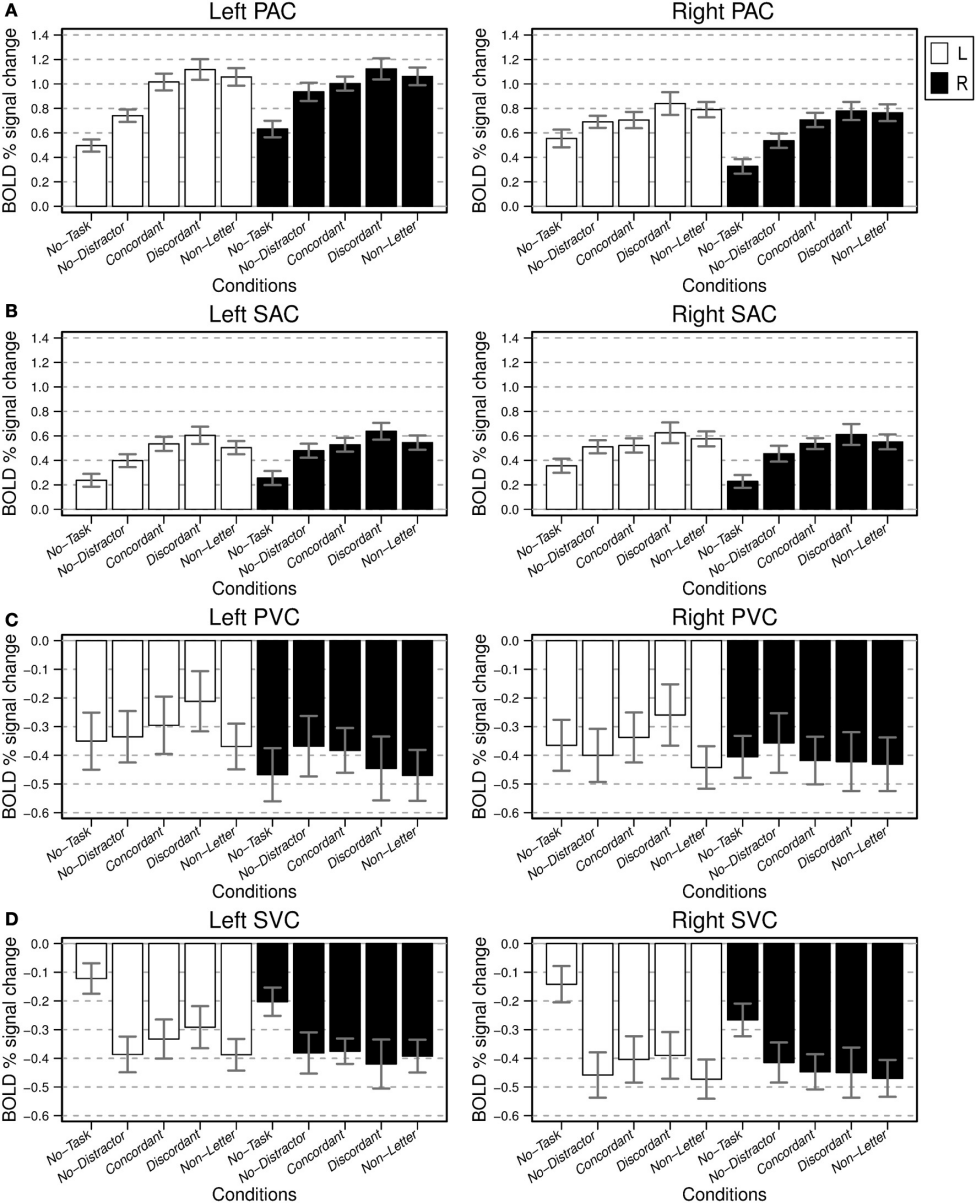
**BOLD percentage signal change relative to baseline for the considered sensory ROIs: PAC (A), SAC (B), PVC (C), and SVC (D).** For each subject the mean fitted responses of all voxels, for each ROI, were determined. The bar plots present average ROI means and respective standard error across subjects, for the attend left (L, white bars) and attend right (R, black bar) ear experimental conditions. Note the different scales for the auditory and visual ROIs, respectively.

Subsequently, the left and right primary and secondary auditory and visual cortices were analyzed further. The BOLD percentage signal change in these sensory ROIs is presented in Figure 6 by means of barplots indicating the mean activation for each combination of ear and condition; ANOVA results are listed in Table 1 as well.

In general, activity in the left PAC was larger than activity in the right PAC. Left PAC and SAC exhibited a highly significant effect of the factor condition; the effect of ear was not significant. For the right PAC, both the effects of ear and condition were significant. For the right SAC there was a significant effect of condition but no significant effect of ear.

We subsequently considered various pairwise comparisons of interest (Table 2). Consistent with the contrasts in Figure 4A, *post-hoc* analysis revealed that for both bilateral PAC and SAC, and both attending L and R, activation to the *No-Task* condition was significantly different from baseline. Furthermore, and also consistent with the contrasts in Figure 4A, in the passive monotic condition (*No-Task*), right PAC activation was stronger when stimuli were presented to the left ear than when presentation was to the right ear (L-*No-Task* vs. R-*No-Task*); this comparison was not significant for left PAC, although a similar trend toward a contralateral preference existed. During the monotic condition with task performance (*No-Distractor*) right PAC activations were stronger when attending the left ear than when attending the right ear (L-*No-Distractor* vs. R-*No-Distractor*). This was only a trend for left PAC. Left and right PAC/SAC activations exhibited a similar pattern: activation during the monotic active task condition (*No-Distractor*) significantly increased relative to passive listening conditions (*No-Task*).

**Table 2:** **Significance of the contrasts of the sensory ROIs, for the Attend Left (L) and Attend Right (R) experimental conditions**.

**Contrasts**	**Attend left (L)**				**Attend right (R)**			
	**L-PAC**	**R-PAC**	**L-SAC**	**R-SAC**	**L-PAC**	**R-PAC**	**L-SAC**	**R-SAC**
**STIMULUS EFFECT**								
*No-Task* vs. baseline	^***^	^***^	^***^	^***^	^***^	^***^	^***^	^***^
**TASK EFFECT**								
No-Distractor vs. No-Task	^***^	^*^	^*^	^*^	^***^	^**^	^**^	^**^
**DISTRACTOR EFFECT**								
Concordant vs. No-Distractor	^***^	n.s.	n.s.	n.s.	n.s.	^**^	n.s.	n.s.
Discordant vs. No-Distractor	^***^	n.s.	^*^	n.s.	^*^	^***^	^*^	n.s.
Non-Letter vs. No-Distractor	^**^	n.s.	n.s.	n.s.	^*^	^***^	n.s.	n.s.
**Contrasts**	**Attend left (L)**				**Attend right (R)**			
	**L-PVC**	**R-PVC**	**L-SVC**	**R-SVC**	**L-PVC**	**R-PVC**	**L-SVC**	**R-SVC**
**STIMULUS EFFECT**								
No-task vs. baseline	^**^	^***^	^*^	n.s.	^***^	^***^	^**^	^***^
**TASK EFFECT**								
No-Distractor vs. No-Task	n.s.	n.s.	^**^	^**^	n.s.	n.s.	^*^	^*^
**DISTRACTOR EFFECT**								
Concordant vs. No-Distractor	n.s.	n.s.	n.s.	n.s.	n.s.	n.s.	n.s.	n.s.
Discordant vs. No-Distractor	n.s.	n.s.	n.s.	n.s.	n.s.	n.s.	n.s.	n.s.
Non-Letter vs. No-Distractor	n.s.	n.s.	n.s.	n.s.	n.s.	n.s.	n.s.	n.s.

Contrasts for the selected families of null-hypotheses were assessed by means of Student t-tests and corrected for multiple comparisons using False Discovery Rate (FDR) criteria, controlled at 0.05 level. Additionally, comparisons were made between corresponding stimuli presented to the left or right ear; these are not listed in the Table, but see the main text for significant results.

* p < 0.05;

** p < 0.01;

*** p < 0.001; n.s., non-significant.

A further increase of signal occurred due to the presence of an irrelevant stream (*Concordant, Discordant, Non-Letter*) relative to the condition without such a stream (*No-Distractor*). This effect was strongest and always significant in PAC on the side contralateral to the task-irrelevant stream (thus, ipsilateral to the attended ear). This increase was significant for the left PAC when attending the right ear but only for *No-Distractor* vs. *Discordant* and *No-Distractor* vs. *Non-Letter* comparisons. Left SAC partially exhibited the same pattern with the following comparisons being significant: L-*No-Distractor* vs. *Discordant* and R-*No-Distractor* vs. R-*Discordant*. For right SAC none of these comparisons reached significance.

Comparisons between the dichotic conditions (*Concordant* vs. *Discordant*; *Concordant* vs. *Non-Letter;* and *Discordant* vs. *Non-Letter*) did not reach significance although the figures show a trend with the most difficult condition (*Discordant)* systematically resulting in the largest mean activation.

The ROIs in the visual cortex showed negative BOLD responses relative to baseline (Figures 6C,D). Left and right primary visual cortex (PVC) showed no significant effect of either condition or ear (Figure 6C). Left and right SVC both showed a significant effect of condition but no significant effect of ear (Figure 6D).

*Post-hoc* analyses revealed for both left and right PVC, and SVC a significant difference between the activation during passive listening conditions and the baseline, except for right SVC, which exhibited only a trend. In contrast with PVC, SVC showed stronger negative responses relative to baseline for each of the four conditions requiring task performance when compared to the No-Task condition. No further comparisons between conditions for left and right SVC reached significance. Comparisons between conditions for left and right PVC were not significant.

## Discussion

This study provides a comparison between fMRI BOLD signal changes resulting from monotic unattended stimulation (*No-Task*), monotic attended stimulation (*No-Distractor*), and dichotic stimulation with one attended and one unattended ear simultaneously (*Concordant, Discordant, Non-Letter*). Widespread areas of the brain were shown to be active for all task conditions. Consistent with previous work, strong BOLD signal changes resulting from monotic stimulation were observed in bilateral AC, responses being largest on the side contralateral to stimulation. We showed that this was the case for both attended task-relevant sound stimuli as well as for unattended task-irrelevant sound stimuli, whether accompanied by task-relevant stimulation of the other ear or not. Moreover, we found that the preferred contralateral activation to task-irrelevant stimuli was strongest for the condition involving stimuli that interfered most strongly with task performance. The activations to two distinct unattended streams were contrasted directly: one from a monotic passive listening condition and the other from a dichotic condition in the presence of a task-relevant stream to the other ear. The latter showed weaker activation in the bilateral AC than the former. Finally, we showed that passive listening is enough to deactivate primary and secondary visual cortex, suggesting that cross-modal inhibition does not require task performance.

### Activation to monotic stimuli

The present study comprised five distinct conditions, per attended ear. The passive listening condition (No-Task) serves as a model for a situation in which a subject is exposed to environmental stimuli but not specifically attending to them since no task is required to be performed. Following monotic task-irrelevant stimulation (*No-Task*), both contralateral and ipsilateral AC were active. Yet, voxel-based analyses showed that the strongest activity was measured in the hemisphere contralateral to the stimulated ear. In agreement was the ROI-based analysis showing that PAC activation was stronger when the stimuli were presented to the contralateral ear. This was particularly significant for the right PAC, whereas left PAC exhibited only a trend. This is in agreement with previously reported results, both in animals (e.g., Rosenzweig, 1951; Hall and Goldstein, 1968; Mrsic-Flogel et al., 2005, 2006; Nelken et al., 2008; Werner-Reiss and Groh, 2008) and humans (e.g., Pantev et al., 1998; Alho et al., 1999; Fujiki et al., 2002; Jäncke et al., 2002; Petkov et al., 2004; Langers et al., 2005; Della Penna et al., 2007; Woods et al., 2009).

Although the stimuli presented during the passive condition were task-irrelevant, we cannot completely exclude that attention may still have been drawn to them. However, given the fast stimulus presentation rate (Alain and Izenberg, 2003), it seems reasonable to assert that a state of sustained and focused attention was absent during this condition, or at least weaker if compared to the other conditions that required task performance. After the session the majority of subjects reported that they had indeed not been performing the task when not instructed to. Furthermore, supporting this view were the ROI BOLD responses: the No-Task condition exhibited weaker activation in the auditory cortices (PAC and SAC) than the No-Distractor task condition. Such task-related attentional enhancement of activity relative to No-Task, during the presentation of the same stimuli, has been previously reported (Grady et al., 1997; Jäncke et al., 1999; Hall et al., 2000).

Conversely, for the four task conditions (No-Distractor, Concordant, Discordant and Non-Letter) focused attention was present. This was firstly confirmed by the behavioral results showing that all subjects performed well above chance level, suggesting that all subjects were engaged during task conditions. Secondly, the No-Distractor vs. No-Task contrast showed activation in motor and premotor cortices, supplementary motor area (SMA) and pre-SMA, and cerebellum. These areas are known to be active during task performance that comprises sensory, cognitive and motor processing (Picard and Strick, 2001; Jäncke et al., 2003; Salmi et al., 2009; Baumann and Mattingley, 2010). In summary, our observations suggest that during passive listening there was no task-related activity and subjects were likely not internally performing the task, while during task conditions subjects were attentively engaged in the one-back task.

### Activation to dichotic stimuli

During three of the five conditions (*Concordant, Discordant* and *Non-Letter*) subjects were presented with both a task-relevant and a task-irrelevant stimulus stream. The introduction of additional task-irrelevant distracting stimuli increased the difficulty of the task. Yet, subjects' mean performance remained above 85% for all conditions with few subjects scoring above 90% for all conditions. Ceiling effects may be present suggesting that the task was not difficult enough, allowing the subjects not only to attend the stimuli in order to perform the task but also to attend the distractors. Still, we were able to show significant differences in task performance between various pairs of conditions (Discordant vs. Concordant, Discordant vs. Non-Letter, Discordant vs. No-Distractor). Thus, we argue that the various distractor types interfered differently with task performance.

Responses resulting from contrasting all distractor conditions against the No-Distractor condition were not found bilaterally (Figures 4C,D). These were strongest in the hemisphere contralateral to the additional distractor stream, ipsilateral to the attended ear. Also, from the ROI analyses AC responses to the addition of a second task-irrelevant stream did not produce significantly stronger responses on the side contralateral to the attended ear, with an exception for the left PAC and SAC. These areas additionally showed significant differences between the No-Distractor condition and some of the dichotic conditions. These results were consistent with the stronger contralateral responses in all monotic conditions. There was an acoustic response increasing the contralateral hemispheric responses due to the task-irrelevant stream presentation, which may be explained by the fact that the No-Distractor condition was monotic while the distractor conditions were dichotic. AC BOLD responses to a dichotic stimulus presentations have been reported to be stronger than those presented monotically (Scheffler et al., 1998). Furthermore, not having a correspondent strong increase in ipsilateral responses may indicate that the already strong contralateral responses to the task-relevant stream could not be significantly elevated by adding a second task-irrelevant stream. This may be attributable to hemodynamic response saturation. BOLD fMRI is not directly sensitive to neural activity, but measures the increased blood flow that follows the increased metabolic demand of activated brain tissue. Because the achievable amount of vascular dilation is limited, BOLD responses tend to saturate at high levels. Such non-linearities would also express themselves as apparent suppression of evoked responses when baseline activity is elevated by the attended sound.

We found stronger activation in the left PAC when compared to the right PAC, in dichotic listening conditions and regardless of the side that is being attended. This can be related to the previously reported phenomenon known as “right ear advantage” (REA) for verbal stimuli in subjects showing left-lateralization for language processing (e.g., Kimura, 1961; Foundas et al., 2006; Della Penna et al., 2007). It has been shown that, behaviorally, attention plays an important role in dichotic listening (Kinsbourne, 1970; Bryden et al., 1983). Correspondingly, neuroimaging studies have showed that the level of activation in the auditory cortex depends on the direction of attention: selective attention directed to one ear increases activation in the auditory cortex contralateral to the attended ear (Alho et al., 1999, 2012; Jäncke et al., 2001, 2003). Thus, it would be expected that when directing attention to the left ear the correspondent contralateral responses, in the right hemisphere, would be strongest when compared to the ipsilateral responses, in the left hemisphere. This results are in agreement with previous research reporting left hemisphere preference for language processing (Damasio and Geschwind, 1984; Giraud et al., 2007).

Activations were different for different distractor types, although ROI-based analyses showed only a trend. Among the dichotic conditions, the strongest activation was measured for the most difficult condition, in both primary and secondary AC. One could argue that this is due to pure bottom-up effects related to the complexity of the auditory scene: two voices speaking different letters (in Discordant), or one voice and one non-voice (in Non-Letter), may require more acoustic processing to be disentangled than two voices speaking the same letter (in Concordant). However, even in the Concordant condition the two streams were spoken by different speakers, one male and one female. Although semantically the same, these were therefore acoustically very different. In particular, with regard to average pitch or spectra, the two Concordant streams were comparably different as the Discordant streams. More generally, for all three of our dichotic conditions the auditory scene consisted of two clearly distinguishable sound sources or auditory objects. This suggests that the differences in activation evoked by the various dichotic distractor conditions were not purely due to the required amount of low-level acoustic processing, but were affected by more high-level functions as well. This may have comprised increased attentional requirements in order to manage greater interference, in accordance with previously reported studies on attentional modulation in the AC (e.g., Jäncke et al., 1999; Petkov et al., 2004; Woods et al., 2009). Given these arguments we conclude that not only bottom-up acoustic mechanisms but also top-down attentional processing was present during dichotic presentations.

Previous work has discussed the influence of attentional load and task difficulty in stimulus processing. While some reported decreased responses with increased task demands (Lavie, 1995; Rees et al., 1997) others reported the opposite relationship (Fockert et al., 2001) or differentiated effects (Alain and Izenberg, 2003; Chait et al., 2012). Lavie et al. (2004) suggested the existence of two types of load: perceptual load and working memory load, with opposite effects (for a review see Lavie, 2005). Recent work (Sabri et al., 2013) showed that increased perceptual load in the attended ear correlates with decreased responses in the auditory cortex to task-irrelevant sounds. This is the opposite from the trend that we observe (increased responses for the most demanding conditions) and furthermore inconsistent with another recent study that did not show any modulatory effect (Murphy et al., 2013). However, our present paradigm differs from Lavie's model in an important regard: we did not vary the perceptual load of the attended stream itself, which remained unchanged over the whole experiment. Instead, we only changed the congruency or the category of the unattended task-irrelevant stream. We surmise that there was an indirect load change that happened through the interference of the different distractors. The most interfering distractor acted to increase the cognitive load of the condition. Thus, differences regarding the nature of the task used might be relevant, considering that Sabri et al. (2013) use a perceptual detection task while the current study requires the participants to perform a cognitive control task, specifically a working memory task. Cognitive control of attentional processes is necessary for minimizing distractor interference, which is the case in the present experiment where one task-relevant stream competes for attention with another task-irrelevant stream. The discrepancy between these studies may therefore be attributed to the observation that working memory load and perceptual load involve different perceptual and cognitive processes (Fockert et al., 2001; Lavie and Fockert, 2005; Dalton et al., 2009).

### Suppressive binaural interaction

To further understand task-irrelevant processing, we addressed the differences between responses to a monotic task-irrelevant stream and responses to a task-irrelevant stream in a dichotic stimulation during simultaneous presentation of a task-relevant stream. We were primarily interested in the neural responses due to the additional unattended distractor stream. To assess the response to an unattended distractor stream, we compared a diotic condition with an attended and an unattended stream to a monotic condition with an attended stream alone. Additionally, we wished to assess whether the presence of the attended stream influences the measured response to the unattended stream. For this purpose, we also measured the neural response to an unattended monotic stream (*No-Task*) compared to a baseline without any streams. Given the responses to an unattended stream in the presence and absence of another attended stream, we could finally assess the interaction between both streams.

The monotic presentation resulted in stronger bilateral auditory cortex activation. Based on previous research it seems reasonable to expect that the two existing ipsilateral pathways are suppressed during dichotic listening, due to dichotic interaction. However, which pathway is being suppressed by the other cannot not be distinguished from our results. We can, however, say that there is evidence of a suppressive interaction mechanism involving the contralateral and the ipsilateral pathways.

Suppressive binaural interaction was proposed in previous studies comparing left and right monotic with dichotic stimulations (e.g., Fujiki et al., 2002; Kaneko et al., 2003). Fujiki et al. (2002) reported suppression of the ipsilateral responses during dichotic stimulation when compared to monotic stimulation, in both hemispheres. The authors discussed this result in terms of existing inhibitory effects present during dichotic stimulation that lead to competition between auditory stimuli. We argue that a similar mechanism occurred in this experiment which requires the processing of two distinct streams: the task-relevant stream, which is supposedly attended, and a task-irrelevant distractor stream that has to be ignored. For left and right presentations, only the contralateral responses to the additional presentation of a task-irrelevant stream (in the presence of a task-relevant stream in the other ear) showed significance, and not the ipsilateral responses to the same stimuli. This can be related with an increase of the ipsilateral responses to the attended stimuli. However, it also suggests that the ipsilateral response of the task-irrelevant stream was suppressed by the stronger contralateral attended task-relevant stream or by an active suppression mechanism of the task-irrelevant stimuli, in agreement with what was suggested in previous research (Alho et al., 1999). Thus, we cannot exclusively argue in favor of the existence of a suppression of the ipsilateral responses of the attended stream. This is an interesting finding which might be correlated with the previously mentioned EEG result showing that the N1 amplitude is reduced in the presence of competing task-irrelevant auditory distractions presented to an unattended ear, when attention is directed to a task-relevant stream simultaneously presented in the other ear (Ahveninen et al., 2011; Ponjavic-Conte et al., 2012).

Although we were limited in the number of conditions due to practical concerns, we concede that other conditions might have been of interest. For example, the inclusion of a passive dichotic listening condition (possibly comprising all three combinations of streams used in this study) would enable a comparison between activation to the presentation of two unattended streams (passive dichotic) with activation to one unattended stream in the presence of another attended stream (active dichotic). It would therefore allow the assessment of the effect of task-relevance on one stream, for instance through top-down attention. Given our primary focus on the unattended stream, we chose to include only the conditions that were required to make the assessments that we present reported on. We nevertheless feel that future studies including these other conditions constitute an important complement to the present work.

### Visual cortex responses

Deactivation of SVC during passive listening relative to the baseline condition was not completely unexpected, however PVC deactivation was. Additionally, the ROI analyses showed that in comparison to PVC, SVC appeared to be more strongly affected by task performance: the No-Distractor vs. No-Task comparison was significantly different for SVC and not for PVC; during passive listening (No-Task) PVC deactivation was stronger than that in SVC. This suggests stronger task-related attentional influences in non-primary visual than primary visual cortex, in agreement with previous studies (Hairston et al., 2008; Mozolic et al., 2008). Moreover, increased task difficulty, with the addition of distractors, did not produce any significant change compared to the active condition with no distractor (No-Distractor), which is different from what has previously been suggested for the SVC (Hairston et al., 2008). Hairston et al. (2008) employed an auditory temporal-order judgment task at different levels of difficulty that were adjusted for each individual's own threshold. This is considered to be a perceptually demanding task. Possibly, the present study employed an easier task requiring less attentional-related resources and consequently a weaker task-difficulty modulatory effect. Additionally, as argued before, differences in the results obtained may reflect distinct neural processes that are task-related, since the present study used a cognitive working memory (as opposed to a perceptual task).

Cross-modal inhibition has been reported in previous studies. In the context of unimodal stimulus presentations, several studies have shown decreased responses in sensory areas that are not classically considered to be relevant to the processing of the presented stimuli (Haxby et al., 1994; Zatorre et al., 1999; Laurienti et al., 2002; Johnson and Zatorre, 2005; Hairston et al., 2008; Salo et al., 2013), although others do not consistently present similar results (for a review see Shulman et al., 1997). In particular, decreased responses to unimodal auditory stimulation have been reported in visual areas, during active conditions requiring auditory sustained attention (Zatorre et al., 1999; Johnson and Zatorre, 2005; Hairston et al., 2008; Mozolic et al., 2008; Salo et al., 2013) and also, although less commonly reported, during passive stimulation (Laurienti et al., 2002; Johnson and Zatorre, 2005). The referred visual related areas were generally confined to higher processing regions (BA19). Interestingly, however, in the present study we show decreased responses not only in higher visual cortex (BA19) but also in the earlier visual processing region in the primary visual cortex (BA17).

We show decreased responses in the primary visual cortex during both auditory active (with or without distractor presence) and passive stimulation (without distractor presence), and with simultaneous increased responses in the auditory cortex. The existence of anatomical connections between auditory and visual areas has been reported before in nonhuman primates (Falchier et al., 2002; Rockland and Ojima, 2003; Clavagnier et al., 2004; Cappe and Barone, 2005). Recently, an interesting study has shown that activation of auditory cortex to passive sound exposure drives synaptic-inhibition in the primary visual cortex, through recruitment of local inhibitory circuitry (Iurilli et al., 2012). Our results for the primary visual cortex are in agreement with the existence of a functional relation between auditory and visual cortex that does not necessarily require attention, and strongly suggest that an automatic sensory processing mechanism occurs within the visual cortices, during acoustic stimulation. Since secondary visual areas seem to be more attentionally modulated than the primary visual, it can be speculated that deactivation of primary sensory areas triggers the (re)allocation of attentional resources within a modality, potentially through the involvement of supramodal areas like frontal and parietal cortices, for further use by the relevant cortices. Future research is necessary to better understand the mechanisms underlying cross-modal interactions.

### Conflict of interest statement

The authors declare that the research was conducted in the absence of any commercial or financial relationships that could be construed as a potential conflict of interest.
